# Yiqi Huoxue Tongluo decoction combined with conventional medicine therapy: effects on restenosis rates and clinical efficacy in patients with coronary heart disease undergoing percutaneous coronary intervention

**DOI:** 10.3389/fcvm.2025.1600189

**Published:** 2025-09-22

**Authors:** Xing Zhao, Lihua Zhang, Zhijie Liu

**Affiliations:** ^1^ Department of Cardiovascular Area Ⅲ, Handan Mingren Hospital, Handan, Hebei, China; ^2^ Department of Anesthesiology, Handan Central Hospital, Handan, Hebei, China

**Keywords:** Yiqi Huoxue Tongluo decoction, conventional medicine therapy, percutaneous coronary intervention (PCI), restenosis (RS), Seattle Angina Questionnaire, cardiac function indexes

## Abstract

**Objective:**

This study aims to investigate the effect of Yiqi Huoxue Tongluo decoction (YHTD) in combination with conventional medicine therapy on restenosis rates and clinical efficacy in patients with coronary heart disease undergoing percutaneous coronary intervention.

**Methods:**

A total of 80 patients with coronary artery disease who underwent PCI between August 2019 and February 2021 were selected, and the patients were assigned to either the control group (*n* = 40) or the observation group (*n* = 40) according to a randomized numerical table. The control group received conventional medicine treatment, while the observation group took YHTD alongside conventional medicine treatment. Coronary angiography was performed after 6 months of treatment to compare the changes in coronary restenosis, TCM syndrome scores, Seattle Angina Questionnaire (SAQ) scores, cardiac function indexes, and related serum biomarkers between the two groups.

**Results:**

After treatment, the TCM syndrome scores of patients in both groups were lower than those before treatment, and the post-treatment TCM syndrome scores of the observation group were significantly lower than those of the control group. Similarly, the SAQ scores of patients in both groups were significantly higher than those before treatment, and the SAQ scores of the observation group were significantly higher than those of the control group. After treatment, both groups showed significant reductions in the LVEDD and LVESD compared with those before treatment, and the LVEDD and LVESD values of the observation group were significantly lower than those of the control group. Similarly, the LVEF and SV of patients in both groups increased compared with those before treatment; however, the LVEF and SV values of the observation group were significantly higher than those of the control group, and the difference was statistically significant. After treatment, hs-CRP, Hcy, and sST2 were significantly reduced in both groups, and the levels of hs-CRP, Hcy, and sST2 in the observation group were significantly lower than those in the control group.

**Conclusion:**

YHTD combined with conventional medicine therapy can significantly reduce the rate of coronary restenosis in post-PCI patients, improve the TCM symptoms, alleviate angina symptoms, enhance cardiac function indexes, and effectively regulate serum biomarker levels, which offers a better choice for the treatment of post-PCI coronary artery disease.

## Introduction

Coronary heart disease (CHD) is one of the most common chronic cardiovascular conditions worldwide, posing a serious threat to human health and life expectancy (1). In recent years, the incidence and mortality rate of CHD have remained high, largely due to an aging population, lifestyle changes, and the accumulation of adverse environmental factors (2–5). The pathological basis of coronary heart disease is coronary atherosclerosis, which can lead to blood vessel narrowing or obstruction. This, in turn, triggers myocardial ischemia and hypoxia and, in severe cases, may lead to myocardial infarction, which can be life-threatening (6).

Percutaneous coronary intervention (PCI), as an effective treatment method, has emerged as a major breakthrough in the treatment of coronary heart disease due to its many advantages, such as minimal trauma, faster recovery, and rapid restoration of myocardial blood flow (7–9). A large number of clinical practices and studies have confirmed that PCI can significantly relieve angina symptoms, effectively reduce the incidence of acute myocardial infarction and other serious cardiovascular events, and greatly improve the quality of life of patients in the near future (10). However, post-PCI revascularization remains a critical problem, affecting the long-term prognosis and quality of life of patients (11, 12). Relevant studies have shown that drug-eluting stent (DES) in-stent restenosis (ISR) occurs in 3%–20% of patients (8, 11). The occurrence of in-stent restenosis (ISR) not only necessitates repeat interventions or surgical bypass surgery, which greatly increases healthcare costs and patient suffering, but also significantly reduces patient survival and quality of life (13).

Currently, conventional medicine mainly adopts comprehensive treatment strategies such as antiplatelet therapy, lipid regulation, and blood pressure and glucose control for the occurrence of ISR after PCI (14–16). Antiplatelet drugs, such as aspirin and clopidogrel, help reduce the risk of restenosis by inhibiting platelet aggregation and reducing thrombosis (14); additionally, statins, which are lipid-regulating agents, play a role in restenosis prevention by lowering lipid levels and stabilizing plaques (17, 18). However, these treatments have obvious limitations. For example, long-term use of antiplatelet drugs can lead to bleeding and other serious adverse reactions, and some patients may also develop drug resistance, which reduces the therapeutic efficacy (19).

In China, traditional Chinese medicine (TCM) is widely used for the prevention and treatment of cardiovascular diseases (20). In Chinese medicine, coronary artery disease belongs to the category of blood stasis syndrome, characterized byblood stasis, phlegm obstruction, qi deficiency, and cold condensation (20, 21). Post-PCI restenosis is thought to be caused by arterial and meridian damage resulting from qi deficiency, blood stasis, and cardiac meridian paralysis (22). Therefore, the principle of benefiting qi and activating blood has become a general treatment method for managing post-PCI restenosis (22, 23). In recent years, with the vigorous development of integrated Chinese and conventional medicine, the study of combining Chinese medicine with conventional medicine for the post-PCI treatment of coronary artery disease has gradually become a hotspot, which is expected to provide a new solution to reduce the rate of post-PCI restenosis and improve clinical outcomes for patients (20, 24).

Yiqi Huoxue Tongluo decoction (YHTD) originates from traditional Chinese medicine theory and has the main effects of benefiting qi, promoting blood circulation, and dredging the collaterals. Modern pharmacological studies have shown that qi-benefiting and blood-activating Chinese herbal compounds can exert anti-atherosclerotic effects by regulating blood lipids, inhibiting platelet activation, suppressing the expression of MCP-1 and NF-κB, and reducing the levels of inflammatory markers (25). Multiple studies have demonstrated that active components of Chinese herbs can improve vascular endothelial function by inhibiting the TLR4/NF-κB signaling pathway (26, 27). Based on this background, this study aims to systematically observe the effects of YHTD in combination with conventional medicine on restenosis rates and clinical efficacy in patients with coronary artery disease undergoing PCI to provide a more reliable basis and reference for clinical treatment.

## Materials and methods

### Study design and subjects

This study was an open-label, randomized controlled trial. The sample size was calculated based on preliminary data indicating a late lumen loss (LLL) difference of *d* = 0.75, with *α* = 0.05 and a power of 0.8, requiring at least 34 patients per group. Considering a 15% dropout rate, 40 patients per group were enrolled. While patient blinding was not feasible due to the nature of the intervention, outcome assessors were blinded to group allocation when evaluating angiographic results and questionnaire scores.

Eighty patients with coronary artery disease who were hospitalized in the Department of Cardiovascular Medicine at our hospital from August 2019 to February 2021 were enrolled. All patients successfully underwent PCI. The patients were assigned to either the control group (*n* = 40) or the observation group (*n* = 40) according to the random number table method. The control group received conventional medicine treatment, while the observation group was given YHTD in addition to conventional therapy. This study was reviewed and approved by the Ethics Committee of Handan Central Hospital, in accordance with the Declaration of Helsinki.

### Inclusion and exclusion criteria

Patients were eligible for inclusion if they met the following criteria: (1) coronary angiography (CAG) suggested coronary artery stenosis, and stent implantation was performed; (2) diagnosis of qi deficiency and blood stasis syndrome according to traditional Chinese medicine, characterized by the presence of at least two main symptoms (chest pain, chest tightness, shortness of breath, fatigue) and two secondary symptoms (dark complexion, purple tongue, thready and hesitant pulse, dark sublingual veins); (3) no cognitive dysfunction and verbal communication disorders; and (4) provided a written informed consent to voluntarily participate in the trial.

Patients were excluded from the study if they met any of the following conditions: (1) presence of acute complications after PCI; (2) underwent repeat PCI due to restenosis after the initial PCI; (3) diagnosed with severe cardiac arrhythmia or had a pacemaker implanted; (4) suffering from serious primary diseases of the liver, kidney, or hematopoietic system or had psychiatric disorders; (5) known allergy to the study drugs; (6) pregnant or lactating women.

Note: Intravascular ultrasound (IVUS) or optical coherence tomography (OCT) was used only for high-risk lesions due to cost constraints and procedural limitations. This study used CAG as the standard method for assessing restenosis, which is acknowledged as a limitation in the Discussion section.

### Treatment methods

The control group received conventional medicine treatment, consisting of oral administration of 100 mg aspirin tablets once daily, 75 mg clopidogrel once daily, and 20 mg atorvastatin calcium tablets once nightly. These oral drugs were taken continuously for 6 months.

In the observation group, on the basis of the conventional medicine treatment, YHTD was administered starting in the same week as the PCI operation and continuing for 6 months. The Chinese herbal formula consisted of astragalus 30 g, *Salvia miltiorrhiza* 20 g, Rhizoma Ligustici chuanxiong 15 g, safflower 10 g, leeches 6 g, dilong 10 g, scorpion 3 g, and *Glycyrrhiza glabra* 6 g. The herbs were decocted twice (20 min each) in 500 ml of water, the filtrates were combined and concentrated to 300 ml, and patients took 150 ml twice daily (bid) for 6 months.

### Clinical assessment

Comparison of coronary restenosis: Vascular restenosis was assessed using coronary angiography. At 6 months post-operation, follow-up coronary angiography was performed, and restenosis was defined as ≥50% stenosis of the inner diameter of coronary vessels. The restenosis rate was calculated as follows: restenosis rate = (number of restenosis cases/total number of cases) × 100%. The following indexes were measured and recorded: minimal lumen diameter (MLD) was defined as the inner diameter of the narrowest part of the coronary lesion; LLL was defined as the difference between the lumen diameter immediately after stent implantation and the lumen diameter at follow-up; late loss index (LLI) was defined as the ratio of late lumen loss to lumen diameter immediately after stent implantation; net lumen gain (NG) was defined as the difference between the lumen diameter after stent implantation and the preoperative stenosis diameter; net lumen gain index (NGI) was defined as the ratio of the net gain to the preoperative stenosis diameter.

TCM syndrome score: The TCM syndrome score is a tool used to quantify the severity of a patient's symptoms by assigning point values based on symptom intensityand adding them together to obtain a total score: 2 points for mild symptoms (slight discomfort with no impact on everyday life); 4 points for moderate symptoms (significant discomfort that partially affects daily life); and 6 points for severe symptoms (severe discomfort that significantly affects daily life). Common symptoms include chest pain, chest tightness, shortness of breath, insomnia, and fatigue. This scoring system was adapted from the State Administration of TCM guidelines (ZY/T 001.1-1994) (28).

Seattle Angina Questionnaire (SAQ): The SAQ is used to assess the symptoms of CHD and their impact on daily life (29, 30). It contains 19 items that can be categorized into five dimensions: physical limitation, anginal stability, anginal frequency, treatment satisfaction, and disease perception. The scoring of the questionnaire is first based on the questions answered by the patients. First, raw scores are calculated for each of the five dimensions, and these raw scores are then converted into the standardized formula to calculate the standard score of each dimension: standard score = (actual score−minimum score)/(maximum score−minimum score) × 100. Each dimension score ranges from 0 to 100, with higher SAQ scores indicating better functioning and better health-related quality of life.

Cardiac function indexes: Color ultrasound was used to compare the cardiac function [left ventricular ejection fraction (LVEF), left ventricular end-diastolic internal diameter (LVEDD), left ventricular end-systolic internal diameter (LVESD), and stroke volume (SV)] in both groups of patients with coronary artery disease before and after treatment.

Serum biomarkers: Serum levels of high-sensitivity C-reactive protein (hs-CRP), homocysteine (Hcy), and soluble suppression of tumorigenicity 2 (sST2) were determined according to the instructions of the ELISA kit. The hs-CRP ELISA kit (XY-1967) was purchased from Shanghai Xinyu Biotechnology Co., Ltd., and the Hcy (JL10022) and sST2 (JL18366) ELISA kits were purchased from Shanghai Jianglai Biotechnology Co. All procedures were performed in strict accordance with the instructions.

### Clinical data collection and follow-up

In the early morning, 5 ml of fasting venous blood was drawn from the elbow of each patient in a collection tube and naturally agglutinated for 30 min at room temperature; after the blood had coagulated, it was centrifuged at 2,000 r/min for 20 min; the upper serum layer was collected and stored at −80°C for future analysis. Information on patients' age, gender, body mass index (BMI), smoking history, alcohol consumption history, family history of coronary heart disease, and comorbidities (hypertension, hyperlipidemia, diabetes mellitus) was collected. In addition, the levels of fasting blood glucose (FBG), total cholesterol (TC), triglyceride (TG), high-density lipoprotein cholesterol (HDL-C), low-density lipoprotein cholesterol (LDL-C), and other markers were measured using an automatic biochemical analyzer (Beijing Plan New Technology Co., Ltd.).

During the 6-month follow-up period, adverse events were recorded. Two patients in the YHTD group reported mild gastric distension; however, no patients discontinued treatment. The average medication adherence rate was 93%.

### Statistical analysis

Data were statistically analyzed and graphed using GraphPad Prism 9.5.0 software (GraphPad Software Inc., San Diego, CA, USA). The Shapiro–Wilk test was used to assess normality of data distribution, and variables conforming to a normal distribution were expressed as mean ± standard deviation. Comparisons between two groups were performed using an independent samples *t*-test, while within-group comparisons (pre- and post-treatment) were performed using a paired samples *t*-test. Count data were expressed as the number of cases and percentages, and comparisons between groups were performed using the chi-square test. All *P* values were two-sided, and the difference was considered statistically significant at *P* < 0.05.

## Results

### Comparison of baseline data between the two groups of subjects

Eighty patients with coronary artery disease who underwent PCI at our hospital from August 2019 to February 2021 were enrolled in this study. Patients were assigned to either the control group (*n* = 40) or the observation group (*n* = 40) according to the method of a randomized numerical table. The control group received conventional medicine treatment, while the observation group was given YHTD alongside conventional medicine. There were no significant differences between the two groups in terms of age, gender, BMI, smoking history, drinking history, family history of coronary heart disease, comorbidities (hypertension, hyperlipidemia, diabetes mellitus), FBG, TG, TC, HDL-C, and LDL-C, among others (all *P*’s > 0.05) (Table 1).

**Table 1:** Comparison of clinical baseline characteristics.

Sports event	Control group (*n* = 40)	Observation group (*n* = 40)	*t*/*χ*^2^	*P*
Age (years)	63.63 ± 10.63	63.78 ± 8.74	0.069	0.945
Sex (m/f)	22/18	22/18	>0.999	0.626
BMI (kg/m^2^）	24.77 ± 2.15	24.97 ± 2.39	0.393	0.695
Smoking history (*n*, %)	22 (55.00%)	25 (62.50%)	0.464	0.496
Drinking history (*n*, %)	20 (50.00%)	22 (55.00%)	0.201	0.654
Family history of coronary heart disease (*n*, %)	3 (7.50%)	4 (10.00%)	0.157	0.692
Comorbidities (*n*, %)				
Hypertension	24 (60.00%)	27 (67.50%)	0.487	0.485
Hyperlipidemia	16 (40.00%)	18 (45.00%)	0.205	0.651
Diabetes	10 (25.00%)	11 (27.50%)	0.065	0.799
FBG (mmol/L)	5.73 ± 1.36	5.928 ± 1.46	0.634	0.528
TC (mmol/L)	4.55 ± 0.805	4.63 ± 0.842	0.421	0.675
TG (mmol/L)	1.39 ± 0.24	1.43 ± 0.27	0.786	0.434
HDL-C (mmol/L)	1.12 ± 0.25	1.20 ± 0.27	1.328	0.188
LDL-C (mmol/L)	2.77 ± 0.47	2.84 ± 0.50	0.621	0.536

BMI, body mass index; FBG, fasting blood glucose; TC, total cholesterol; TG, triglyceride; HDL-C, high-density lipoprotein cholesterol; LDL-C, low-density lipoprotein cholesterol. The Shapiro–Wilk test was used to assess the normality of data distribution, and variables conforming to a normal distribution were expressed as mean ± standard deviation; comparisons between two groups were performed using an independent samples *t*-test. Count data were expressed as the number of cases and percentages, and comparisons between groups were performed using a chi-square test. The difference was considered statistically significant at *P* < 0.05.

### Comparison of coronary restenosis in two groups

**Figure 1:**
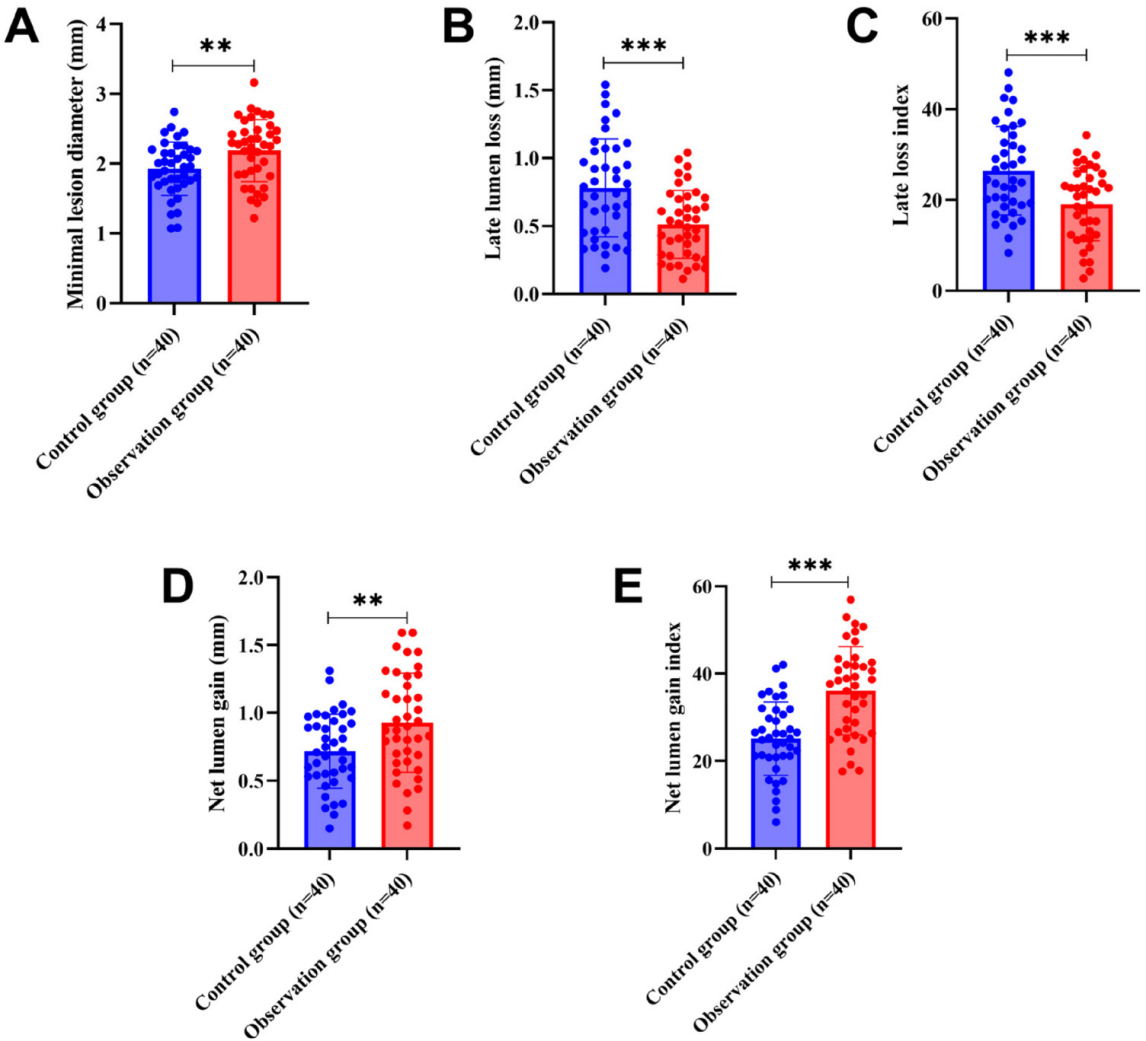
Comparison of coronary restenosis between the two groups of patients (**A**: MLD, **B**: LLL, **C**: LLI, **D**: NG, **E**: NGI). MLD, minimal lumen diameter; LLL, late lumen loss; LLI, late loss index; NG, net lumen gain; NGI, net lumen gain index.

We compared the coronary restenosis in two groups of patients, and the results showed that after 6 months of treatment, 10 cases of restenosis occurred in the control group and 3 cases of restenosis occurred in the observation group, and the rate of restenosis in the observation group (7.50%) was significantly lower than that in the control group (25.00%), and the difference was statistically significant (*χ*² = 4.501, *P* = 0.034). In addition, compared with the control group, MLD, NG, and NGI were significantly larger, and LLL and LLI were significantly lower in the observation group (all *P's* < 0.05), as shown in Figure 1.

### Comparison of TCM syndrome scores between the two groups of patients

**Figure 2:**
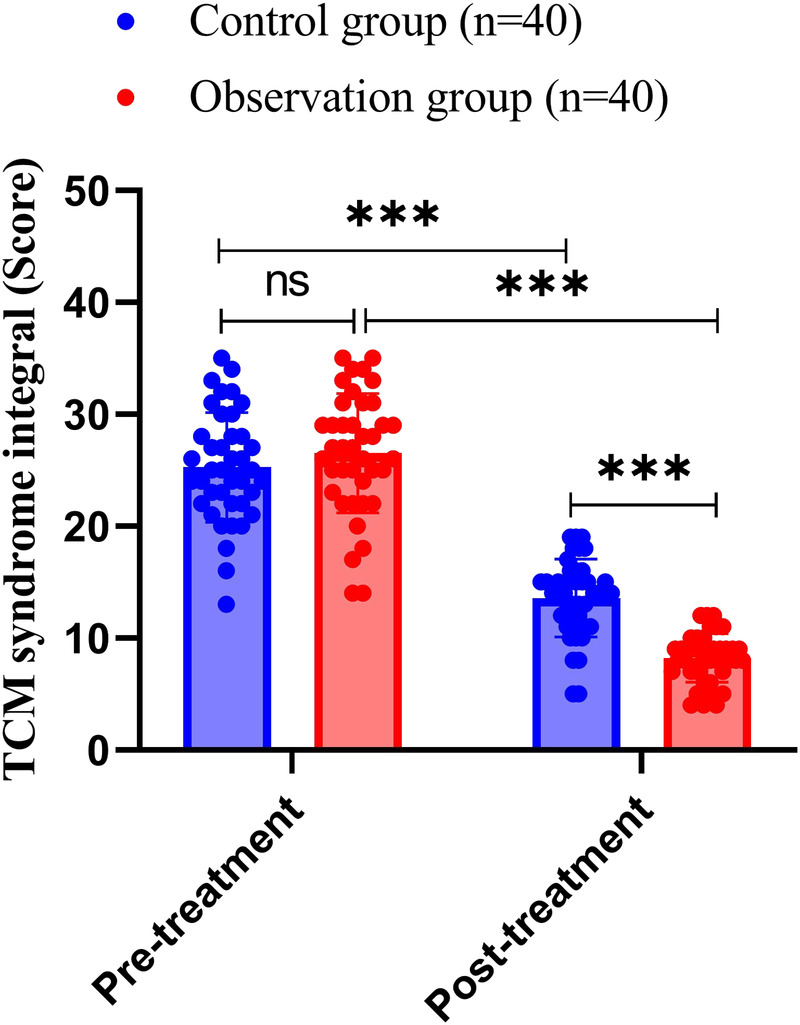
Comparison of TCM syndrome scores between the two groups of patients.

We compared the TCM syndrome scores of the two groups of patients, and the results showed that before treatment, the TCM syndrome score of the control group was 25.25 ± 4.88, while that of the observation group was 26.53 ± 5.32; the difference between the two groups was not statistically significant (*P* = 0.268). After treatment, the TCM syndrome score of the control group was 13.58 ± 3.47, while that of the observation group was 8.23 ± 2.17; both groups showed significantly lower TCM syndrome scores than those before the treatment (both *P* < 0.001), and the post-treatment TCM syndrome scores of the observation group were significantly lower than those of the control group (all *P*’s < 0.001), as shown in Figure 2.

### Comparison of the Seattle Angina Questionnaire scores between the two groups

The SAQ was used to assess the CHD symptoms and their impact on daily life. It was found that there were no statistical differences between the two groups of patients across all SAQ dimensions before treatment (physical limitation, anginal stability, anginal frequency, treatment satisfaction, and disease perception) (all *P*’s > 0.05); after 6 months of treatment, SAQ scores of the patients in both groups were significantly higher than those before treatment (all *P*’s < 0.001). In addition, the post-treatment SAQ scores of the observation group were significantly higher than those of the control group (all *P*’s < 0.05) (Table 2).

**Table 2:** Comparison of Seattle Angina Questionnaire scores between the two groups of patients.

Sports event	Timing	Control group (*n* = 40)	Observation group (*n* = 40)	*t*	*P*
Physical limitation (score)	Pre-treatment	47.60 ± 12.28	49.88 ± 11.33	0.861	0.392
	Post-treatment	63.25 ± 10.13	70.18 ± 11.44	2.866	0.005
	*t*	6.213	7.297		
	*P*	<0.001	<0.001		
Anginal stability (score)	Pre-treatment	34.18 ± 11.22	35.93 ± 12.18	0.668	0.506
	Post-treatment	54.98 ± 13.24	62.75 ± 12.04	2.748	0.008
	*t*	7.941	9.618		
	*P*	<0.001	<0.001		
Anginal frequency (score)	Pre-treatment	55.10 ± 9.948	57.25 ± 12.69	0.843	0.402
	Post-treatment	70.50 ± 9.769	78.60 ± 10.84	3.511	0.001
	*t*	9.212	7.602		
	*P*	<0.001	<0.001		
Treatment satisfaction (score)	Pre-treatment	61.48 ± 10.08	63.23 ± 9.60	0.795	0.429
	Post-treatment	73.55 ± 8.71	78.15 ± 8.98	2.326	0.023
	*t*	5.525	7.310		
	*P*	<0.001	<0.001		
Disease perception (score)	Pre-treatment	42.35 ± 8.97	41.38 ± 7.31	0.533	0.596
	Post-treatment	55.98 ± 11.82	61.50 ± 12.28	2.050	0.044
	*t*	6.302	8.962		
	*P*	<0.001	<0.001		

The Shapiro–Wilk test was used to assess the normality of data distribution, and variables conforming to a normal distribution were expressed as mean ± standard deviation; comparisons between the two groups were performed by an independent samples *t*-test, and a paired *t*-test was used to evaluate changes before and after treatment. The difference was considered statistically significant at *P* < 0.05.

### Comparison of cardiac function indexes before and after treatment in two groups

We compared LVEF, LVEDD, LVESD, and SV between the two groups. The results showed no statistical differences in LVEF, LVEDD, LVESD, and SV between the two groups before treatment (*P* > 0.05); after 6 months of treatment, LVEDD and LVESD were significantly reduced in both groups compared to pre-treatment values, and the reductions were were more pronounced in the observation group than in the control group (both *P*’s < 0.05). Additionally, LVEF and SV significantly increased in both groups after treatment, with the observation group showing greater improvements in LVEF and SV than the control group, and the differences were statistically significant (all *P*'s < 0.05) (see Table 3).

**Table 3:** Comparison of cardiac function indexes before and after treatment in the two groups of patients.

Sports event	Timing	Control group (*n* = 40)	Observation group (*n* = 40)	*t*	*P*
LVEF (%)	Pre-treatment	47.63 ± 5.34	48.73 ± 5.37	0.919	0.361
	Post-treatment	51.60 ± 3.44	56.40 ± 5.59	4.626	<0.001
	*t*	3.994	6.415		
	*P*	<0.001	<0.001		
LVEDD (mm)	Pre-treatment	55.18 ± 4.31	55.43 ± 4.60	0.251	0.803
	Post-treatment	51.50 ± 3.44	49.25 ± 2.25	3.465	<0.001
	*t*	4.064	7.808		
	*P*	<0.001	<0.001		
LVESD (mm)	Pre-treatment	33.58 ± 2.51	33.23 ± 2.68	0.604	0.548
	Post-treatment	30.25 ± 2.26	29.23 ± 2.02	2.138	0.036
	*t*	6.092	6.571		
	*P*	<0.001	<0.001		
SV (V/mL)	Pre-treatment	58.03 ± 2.88	58.93 ± 2.86	1.403	0.165
	Post-treatment	67.18 ± 4.56	70.68 ± 5.84	2.989	0.004
	*t*	9.869	11.620		
	*P*	<0.001	<0.001		

LVEF, left ventricular ejection fraction; LVEDD, left ventricular end-diastolic dimension; LVESD, left ventricular end-systolic dimension; SV, stroke volume. The Shapiro–Wilk test was used to assess the normalilty of data distribution, and variables conforming to a normal distribution were expressed as mean ± standard deviation; an independent samples *t*-test was used for comparison between the two groups, and a paired *t*-test was used to evaluate changes before and after treatment. The difference was considered statistically significant at *P* < 0.05.

### Comparison of serum biomarkers between the two groups of patients

**Figure 3:**
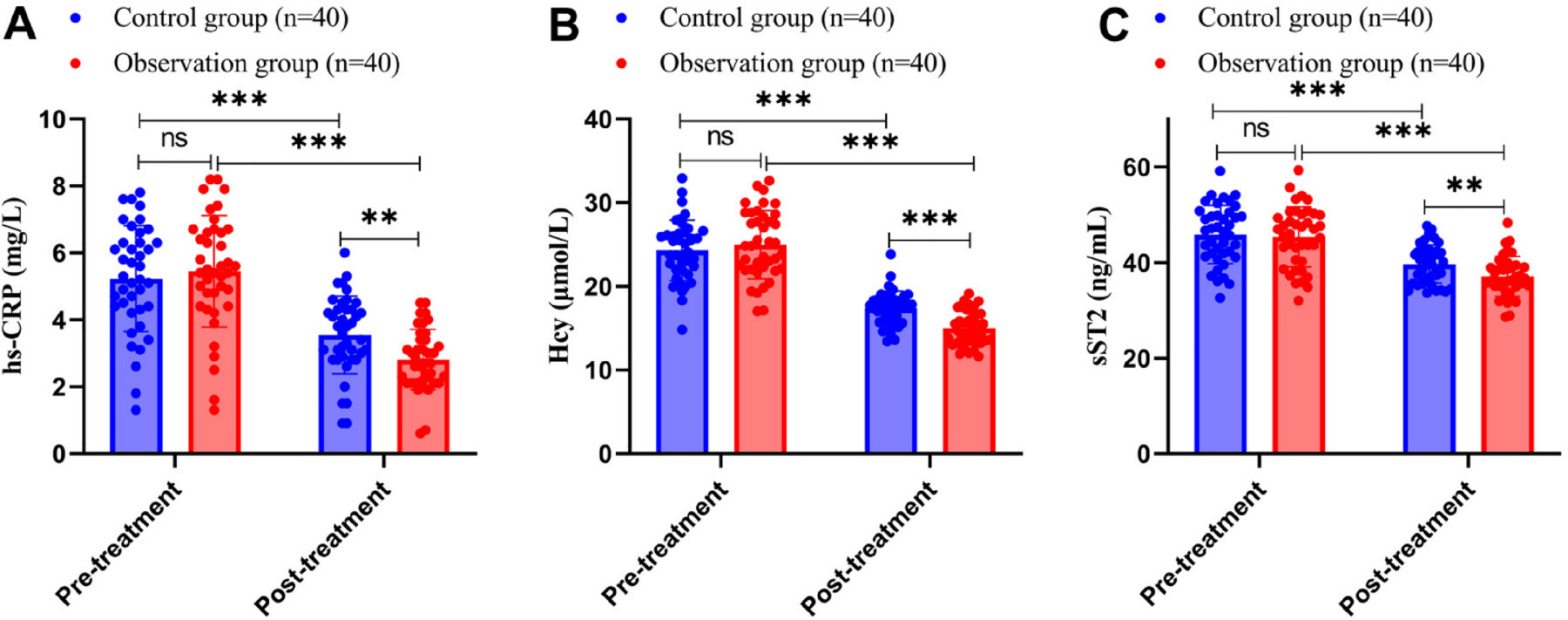
Comparison of serum biomarkers between the two groups of patients (**A**: hs-CRP, **B**: Hcy, **C**: sST2). hs-CRP, high-sensitivity C-reactive protein; Hcy, homocysteine; sST2, soluble suppression of tumorigenicity 2.

Previous studies have reported that elevated levels of hs-CRP and Hcy are associated with a higher risk of ISR after PCI in patients with coronary artery disease (31–33). In addition, elevated levels of sST2 are strongly associated with adverse cardiovascular outcomes after PCI (34). Therefore, we determined serum levels of hs-CRP, Hcy, and sST2 by ELISA, and the results showed no statistically significant differences in the levels of hs-CRP, Hcy, and sST2 between the two groups of patients before treatment (all *P*’s > 0.05). After treatment, the serum hs-CRP level in the control group was 3.55 ± 1.17 mg/L, the Hcy level was 17.41 ± 2.01 μmol/L, and the sST2 level was 39.67 ± 3.98 ng/mL; in contrast, the serum hs-CRP level in the observation group was 2.81 ± 0.90 mg/L, the Hcy level was 15.03 ± 2.07 μmol/L, and the sST2 level was 37.10 ± 4.19 ng/mL. These results indicate that hs-CRP, Hcy, and sST2 levels were significantly reduced in both groups after treatment (all *P*’s < 0.001), with the reductions in the observation group being significantly lower than those in the control group after treatment (all *P*’s < 0.01), as shown in Figure 3. Additional atherosclerosis-specific markers, including ox-LDL and Lp-PLA2, were also measured, showing consistent intergroup differences (Supplementary Table S1).

The Shapiro-Wilk test was used to test for normal distribution, and measurements conforming to normal distribution were expressed as mean ± standard deviation. Independent samples *t*-test was used for comparison between the two groups, and paired *t*-test was used before and after treatment. Differences were considered statistically significant at *P* < 0.05. ns indicates *P* > 0.05, ** indicates *P* < 0.01, and *** indicates *P* < 0.001.

## Discussion

Coronary heart disease is a common chronic cardiovascular condition and remains a serious threat to human health (35). Although PCI has brought a major breakthrough in the treatment of coronary heart disease, post-operative restenosis continues to affect the long-term prognosis of patients (36, 37). In this study, we compared the effects of conventional medicine treatment alone versus YHTD combined with conventional medicine on post-operative PCI patients with coronary artery disease, and the results showed that the combination therapy offered significant advantages in several aspects.

In terms of coronary restenosis, the observation group exhibited a significantly lower restenosis rate than the control group; additionally, the observation group showed increased values of MLD, NG, and NGI and decreased values of LLL and LLI. This suggests that YHTD combined with conventional medicine therapy can effectively inhibit endothelial proliferation, maintain vascular patency, and reduce the risk of restenosis (20, 38). From the perspective of Chinese medicine theory, coronary heart disease belongs to the category of blood stasis syndrome, characterized by blood stasis, phlegm obstruction, qi deficiency, and cold condensation; post-operative restenosis after PCI is believed to be associated with qi deficiency, blood stasis, and cardiac meridian paralysis (22). In YHTD, astragalus tonifies qi and elevates yang; danshen, chuanxiong, and safflower promote blood circulation and remove blood stasis; leech, dilong, and scorpion penetrate the channels to alleviate pain; and *Glycyrrhiza* tonifies the medicines to work together to activate qi and blood and penetrate the channels to alleviate pain (39, 40). Modern pharmacological studies have also confirmed that Chinese medicines with blood-activating and blood-stasis-eliminating properties exert effects such as antiplatelet aggregation, improvement of microcirculation, and inhibition of the proliferation of smooth muscle cells (40, 41). From the perspective of integrative medicine, the concept of qi deficiency in TCM corresponds to endothelial dysfunction and impaired energy metabolism at the cellular level, while blood stasis reflects the pathological states such as increased platelet aggregation, inflammation, and microcirculatory disturbances. Similarly, the meridian blockage concept aligns with vascular stenosis and impaired blood flow.

The therapeutic effects of YHTD can be attributed to its key bioactive components. Astragaloside IV, the main active component of astragalus, exhibits anti-inflammatory and endothelial protective effects by inhibiting the TLR4/NF-κB signaling pathway, thereby reducing the expression of inflammatory cytokines (IL-6, TNF-α) and adhesion molecules (VCAM-1, ICAM-1) while enhancing NO production (42). Tanshinone IIA protects against oxidative stress and inhibits the proliferation and migration of VSMCs, contributing to plaque stabilization (43). Tetramethylpyrazine exerts antiplatelet and anti-inflammatory effects via inhibition of the P38 MAPK and NF-κB signaling pathways (44). These components act synergistically to inhibit phenotypic transformation of VSMCs from a contractile to a synthetic state and suppress neointimal formation by downregulating PDGF and TLR4/NF-κB signaling pathways (45), which may explain the observed reductions in inflammatory markers (hs-CRP, Hcy, sST2) and the decreased restenosis rate in our study. Our findings are consistent with previous studies. For example, Mao et al. (40) reported a similar reduction in restenosis rates in post-PCI patients treated with Tongxinluo capsules. However, our study showed a more pronounced improvement in cardiac function parameters.

TCM, with its multi-component, multi-target, and holistic characteristics, plays an active and vital role in the prevention and treatment of various cardiovascular diseases in China, and it offers significant advantages in stabilizing the condition and improving the quality of life of patients (46). TCM has been recognized as a potential approach for relieving symptoms and improving quality of life in patients with cardiovascular disease (21). The SAQ is the most commonly used tool for assessing comprehensive and sensitive changes in the quality of life of cardiac patients (47). In this study, the TCM syndrome scores and SAQ scores improved after treatment; specifically, TCM syndrome scores of both groups decreased after treatment, with the observation group exhibiting lower scores than the control group; after 6 months of treatment, the scores of all SAQ dimensions increased in both groups, with the observation group exhibiting higher scores than the control group. These findings suggest that combination therapy effectively alleviates angina symptoms, improves TCM symptoms, and enhances the quality of life of patients (48, 49).

It has been reported that herbal formulas containing astragalus, or herbal astragalus alone, can enhance myocardial contractility and improve cardiac diastolic function (50, 51). In addition, shenmai injection may improve cardiac function in patients with chronic heart failure (CHF) through its anti-apoptotic effects, antioxidant activity, anti-inflammatory properties, and improvement of myocardial metabolism (52). Cardiac function indexes showed that LVEF and SV increased, while LVEDD and LVESD decreased in both groups after treatment, and the degree of improvement was better in the observation group than in the control group. These findings indicate that the combination therapy can effectively improve cardiac function and enhance myocardial contractility. This may be due to the synergistic effect of Chinese medicine and conventional medicine in improving myocardial blood perfusion and inhibiting myocardial remodeling, thus enhancing the pumping ability of the heart (53).

hs-CRP is a highly sensitive indicator of inflammatory response, and a decrease in its level reflects a reduction in the degree of inflammation in the body. Inflammation plays a key role in the development and progression of coronary artery disease, and elevated serum levels of hs-CRP have been significantly associated with the occurrence of restenosis (ISR) after coronary intervention (31, 54). In addition, studies have reported that homocysteine (Hcy) levels are positively correlated with the severity of restenosis after coronary intervention and can be used as an important biomarker for predicting the severity of restenosis (55, 56). Meanwhile, sST2 has been identified as an important indicator for assessing the prognosis of cardiovascular disease after PCI (34). The results of serum biomarker analysis showed that YHTD combined with conventional medicine therapies more effectively reduced the levels of hs-CRP, Hcy, and sST2, thus providing new ideas and methods for the post-PCI treatment of patients with coronary artery disease.

However, this study has some limitations. The relatively small sample size of only 80 patients may not comprehensively capture the diversity of patient characteristics, and there is a sampling error, so the representativeness and generalizability of the study results need to be improved. With a follow-up period of only 6 months, it is difficult to accurately assess the long-term efficacy and safety of interventions for post-PCI restenosis, which is a problem with a long-term dynamic process, because the observation period is relatively short. Additionally, this study used an open-label design in which patient blinding was not feasible; however, outcome assessors were blinded. The use of coronary angiography, instead of more sensitive techniques like IVUS or OCT, may have limited the accuracy of restenosis assessment. Future studies can be improved in the following aspects: by expanding the sample size and conducting multi-center, large-sample clinical studies to enhance the reliability and representativeness of the results; by extending the follow-up period to observe the long-term efficacy and safety of the combination therapy; and by incorporating advanced imaging techniques like IVUS or OCT to achieve a more accurate assessment of vascular changes.

Future research directions should include: (1) the application of multi-omics approaches to identify biomarkers predicting treatment response; (2) systematic investigations of specific bioactive compounds and their synergistic effects using network pharmacology; (3) mechanistic studies using advanced molecular techniques such as single-cell RNA sequencing to understand cell-specific responses; and (4) the development of standardized quality control methods for TCM preparations.

## Conclusion

In conclusion, YHTD combined with conventional medicine therapy demonstrates significantly better outcomes than conventional medicine therapy alone in patients undergoing PCI for coronary artery disease; the combination therapy more effectively improves coronary restenosis, reduces TCM syndrome scores, alleviates angina symptoms, enhances cardiac function, and lowers the levels of related serum biomarkers. These findings suggest that YHTD may serve as an adjuvant therapy for post-PCI management. However, to further validate these results and establish its role in clinical practice, multi-center studies with larger sample sizes and advanced imaging techniques (IVUS/OCT) are needed.

## Data Availability

The original contributions presented in the study are included in the article/Supplementary Material, further inquiries can be directed to the corresponding author.
