# Supplementary material for: Yiqi Huoxue Tongluo decoction combined with conventional medicine therapy: effects on restenosis rates and clinical efficacy in patients with coronary heart disease undergoing percutaneous coronary intervention
Source: Front Cardiovasc Med. 2025 Sep 22;12:1600189. doi: 10.3389/fcvm.2025.1600189 (PMC12498481; doi:10.3389/fcvm.2025.1600189)
Supplement: Supplementary file 1 [file Table1.docx]

**Supplementary Table S1: Additional atherosclerosis-specific biomarkers**

**Table S1. Comparison of ox-LDL and Lp-PLA2 levels between the two groups before and after treatment**

| **Biomarkers** | **Time point** | **Control group (n=40)** | **Observation group (n=40)** | ***t*** | ***P*** |
| --- | --- | --- | --- | --- | --- |
| **ox-LDL (U/L)** |  |  |  |  |  |
|  | Pre-treatment | 142.35±18.76 | 145.28±20.14 | 0.675 | 0.502 |
|  | Post-treatment | 118.62±16.23 | 98.47±14.85 | 5.781 | <0.001 |
|  | *t* | 8.234 | 11.962 |  |  |
|  | *P* | <0.001 | <0.001 |  |  |
| **Lp-PLA2 (ng/mL)** |  |  |  |  |  |
|  | Pre-treatment | 287.43±42.58 | 291.67±45.31 | 0.432 | 0.667 |
|  | Post-treatment | 235.78±38.42 | 198.35±32.16 | 4.726 | <0.001 |
|  | *t* | 7.156 | 10.438 |  |  |
|  | *P* | <0.001 | <0.001 |  |  |

**Note:** ox-LDL: oxidized low-density lipoprotein; Lp-PLA2: Lipoprotein-associated phospholipase A2. The Shapiro-Wilk test was used to test for normal distribution, and measurements conforming to normal distribution were expressed as mean ± standard deviation. Independent samples t-test was used for comparison between the two groups, and paired t-test was used for comparison before and after treatment within groups. The difference was considered statistically significant at P < 0.05.
